# Factors Associated with the Acquisition and Severity of Gestational Listeriosis

**DOI:** 10.1371/journal.pone.0013000

**Published:** 2010-09-27

**Authors:** M. Mitsu Suyemoto, Patricia A. Spears, Terri S. Hamrick, Jill A. Barnes, Edward A. Havell, Paul E. Orndorff

**Affiliations:** 1 Department of Population Health and Pathobiology, College of Veterinary Medicine, North Carolina State University, Raleigh, North Carolina, United States of America; 2 Department of Pharmaceutical Sciences, School of Pharmacy, Campbell University Buies Creek, Buies Creek, North Carolina, United States of America; 3 Department of Biomedical Sciences, College of Veterinary Medicine, North Carolina State University, Raleigh, North Carolina, United States of America; East Carolina University School of Medicine, United States of America

## Abstract

Gravid mammals are more prone to listeriosis than their nongravid counterparts. However, many features of the disease in gravid animals are not well defined. We determined, in mice, that increased susceptibility to lethal infection following oral inoculation begins surprisingly early in pregnancy and extends through embryonic development. Pregnancy did not demonstrably increase the spread of listeriae from the intestine to the liver and spleen in the initial 96 h period post inoculation. Consequently, it appeared that gravid animals were competent to contain an enteric infection, but in those instances where escape did occur, a lethal outcome was more likely. Interestingly, colonic colonization level and prevalence, measured 96 h post inoculation, was significantly higher in gravid individuals. In terms of human risk factors for listeriosis, our results suggest that the window of listeriosis susceptibility afforded by pregnancy may be open longer than previously appreciated. Our results also suggest that while gravid animals are competent to contain an enteric infection, enteric carriage rate may be more of a factor in defining disease incidence than previously considered.

## Introduction

Common features of listeriosis are (i) ingestion of contaminated food (ii) colonization of the intestine (iii) intestinal translocation (iv) replication in the liver and spleen, and (v) either the resolution of infection or the hematogenous spread to other organs resulting in a systemic infection [1]. Pregnancy greatly increases the risk of listeriosis in virtually all mammalian species [2], [3]. Approximately thirty-three percent of clinically documented cases of human listeriosis are in pregnant women [4] and pregnant women constitute approximately 60% of all cases (male and female) aged 10 to 40 years [5].

Despite the well-known association of listeriosis with pregnancy, there is incomplete agreement on how pregnancy increases disease risk. Case studies suggest that the gravid host is most susceptible to listeriosis during the later stage of pregnancy. Indeed, with isolated exceptions [6], disease presentation correlates well with a depression in the host's cell- mediated immunity late in gestation [7], [8]. However, early pregnancy terminations (i.e., spontaneous abortions at the embryonic stage rather than at the later fetal stage) are typically not analyzed microbiologically. The gravid host may also display fewer symptoms of listeriosis in early pregnancy or attribute mild symptoms to more routine discomforts (*e.g.*, morning sickness). Experimental studies in mice, in which the timing of impregnation and initiation of listeriosis by oral inoculation can be tightly controlled [9], indicate that gravid animals are prone to disease earlier than the fetal development stage [10], [11]. However, a systematic study to identify the gestational stage at which increased susceptibility first manifests itself has not been done.

In this communication, we report that increased maternal susceptibility to lethal listeriosis begins very early in gestation and extends through embryonic development. Mice at the fetal stage of pregnancy recovered some resistance. Pregnancy did not increase liver and spleen infectivity (measured soon after oral inoculation). However, gestation did increase listerial prevalence and load in the colon throughout gestation.

## Results

### LD_50_ determination at progressive gestational stages

**Figure 1:**
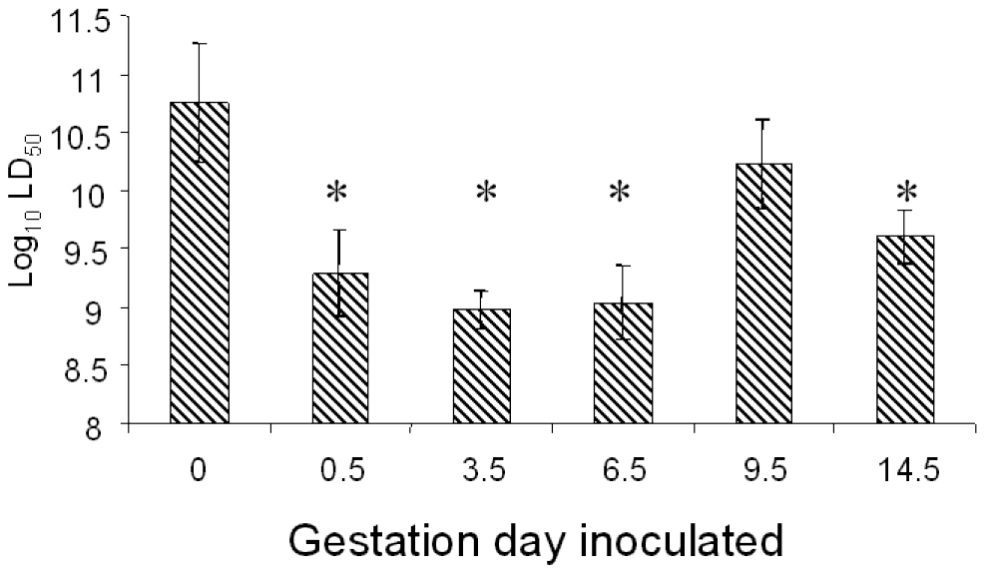
Fifty percent lethal dose (LD_50_) determinations of mice inoculated at different gestational stages. Striped bars indicate the log_10_ colony forming units (cfu) required to produce death in 50% of the inoculated animals. Mice were inoculated and observed as described in the text. At least three groups of animals with three different listerial doses were used to determine the LD_50_. Groups consisted of not fewer than 4 mice. Error bars denote standard error of the mean. Probit analysis was used to determine statistical differences between groups. Asterisks denote values that were statistically different from the nongravid group (denoted by zero gestational days).

The LD_50_ values determined from orally inoculated nongravid and gravid mice (**Fig. 1**) revealed that the LD_50_ was significantly lower at 0.5 gestational days (gd) and reached its apparent nadir (*ca.*, 100-fold less than nongravid) between 3.5 and 6.5 gd. The LD_50_ increased at 9.5 gd to a value indistinguishable from the nongravid LD_50_ before decreasing at 14.5 gd at which time the LD_50_ was *ca.* 10-fold lower than that for nongravid mice.

### Listerial infectivity at progressive gestational stages

**Figure 2:**
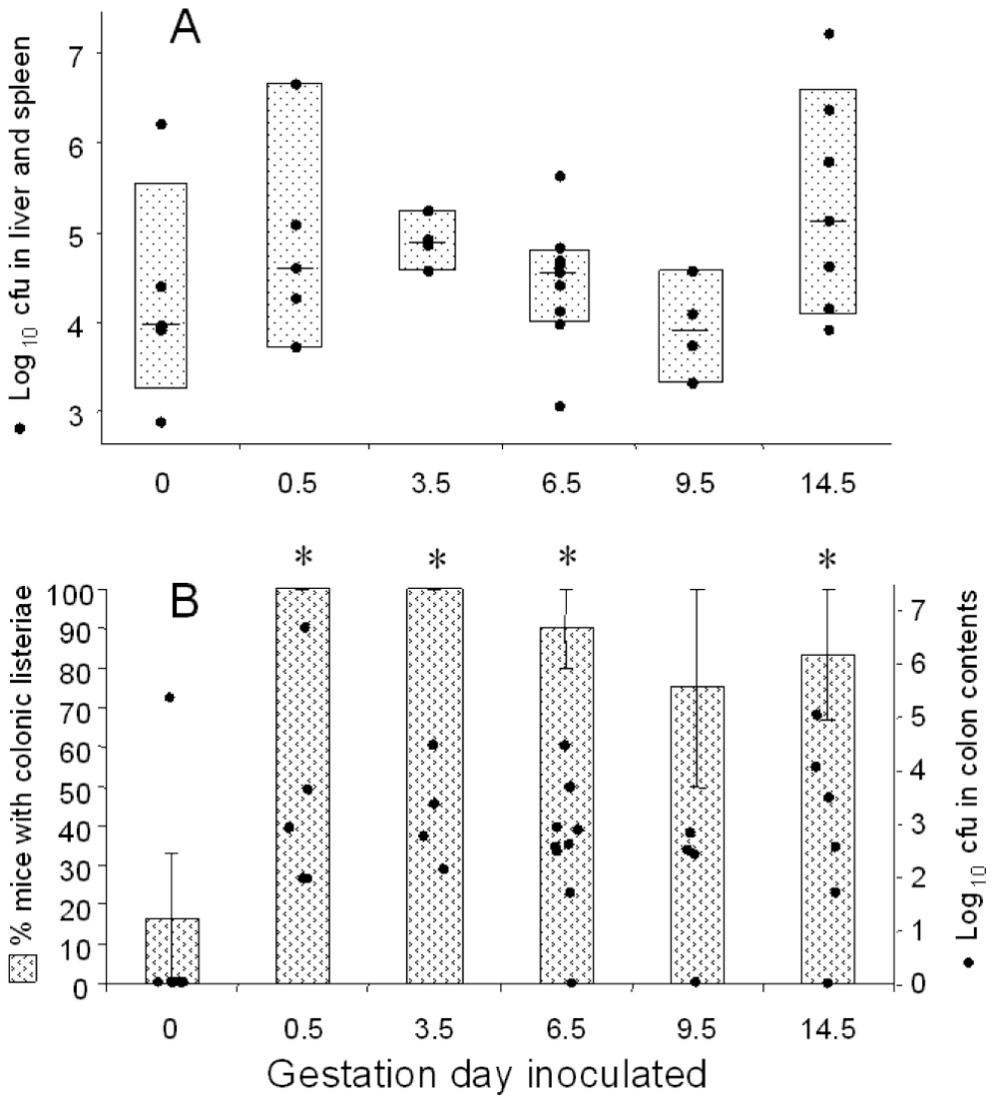
Listerial organ and colonic colony counts in mice inoculated at different gestational stages. Panel A illustrates listerial cfu recovered from the liver and spleen of each pregnant and nongravid (zero gestational day) mouse (denoted by a single dot) at 96 h post inoculation. The stippled rectangles denote the 95% confidence interval for each group. The median colonization level of each group is denoted by a horizontal line. Infectivity data was analyzed using the Mann-Whitney rank sum test. There were no gravid groups that were statistically distinguishable from the nongravid group. Panel B illustrates colonic colonization. Each dot represents an individual mouse. Stippled bars indicate the percentage of mice (left hand ordinate) with detectable colonic cfu when sacrificed at 96 h post inoculation. Error bars denote the standard error of the mean. Asterisks denote groups that are statistically different from the nongravid group as determined using Fisher's exact test. Colonic cfu from the equivalent of 2 fecal pellets from the large intestine of each mouse are denoted by a dot with values listed on the right hand ordinate. In cases where colonic cfu were recovered, the log_10_ value is listed. Mice with undetectable colonic cfu were recorded as zeros.

The ability of gravid and nongravid mice to limit the translocation of listeriae from the intestine to the liver and spleen was assessed 96 h post inoculation at progressive stages of gestation by examining the cfu in the colon contents and in the liver and spleen. We found that gestational stage had no demonstrable effect upon the average level of liver and spleen colonization (**Fig. 2A**). However, there was a significant and dramatic increase in the proportion of gestating mice carrying listeriae in the colon soon after impregnation (**Fig. 2B**). This same increase in listerial prevalence was registered as statistically significant if colonic listerial cfu per mouse (Fig. 2B) were averaged within groups and compared.

## Discussion

Despite the well known susceptibility to listeriosis during pregnancy, there is incomplete agreement on how pregnancy increases listeriosis risk. The generalized suppression of immunity in gravid animals late in gestation is often cited as a factor in increased disease susceptibility [10], [12]. Consequently, most experimental studies that examine the effect of pregnancy on listerial pathogenesis have employed mice at a late stage in their 18.5 gestational period, typically 14.5 gd. However, a number of reports indicate that mice become more prone to infection well in advance of that gestational stage (reviewed in Orndorff et al.[1]). Most recently, Hamrick et al. [11] showed a dramatic increase in susceptibility to infection at 7.5 gd. In the present report, we orally inoculated mice at different stages of pregnancy and determined when increased susceptibility to lethal listerial infection occurred.

Our results indicate that susceptibility to lethal infection occurs very early in gestation. Even animals inoculated at 0.5 gestational days showed a significant increase in susceptibility. Lethality peaked in animals inoculated at 3.5 and 6.5 gd when embryonic development is occurring rapidly. By the time embryonic development ceased (*ca.* 9.5 gd) and fetal maturation commenced, lethality decreased somewhat.

The strikingly early pregnancy stage (0.5 gd) at which susceptibility increased, seemed to eliminate generalized immunological suppression as the cause of the increased lethality. However, to examine this assumption we determined the ability of gravid animals to initially respond to listerial infection by measuring the level of liver and spleen colonization soon after inoculation of a low listerial dose. The short time interval between inoculation and organ harvest and the low inoculating dose employed largely eliminated complicating intrauterine infections in gravid individuals. Only a small subset of gravid individuals with liver and spleen colonization develop intrauterine infections [11], [13], [14]. Nevertheless, such infections can serve as a source for reseeding the liver and spleen [15]. Consequently, intrauterine cfu were monitored. We found that comparatively few mice (4 of the 29 gravid animals examined) had uterine infections at 96 h post inoculation (10^2^ to 10^6^ cfu per organ) and their small representation did not affect the conclusions derived from statistical analysis of liver and spleen cfu: Listerial burdens in the liver and spleen, in gravid and nongravid mice were indistinguishable. This result indicated that the initial immune response of a gravid mouse was similar to that of a nongravid mouse. There was, however, a pronounced increase in the persistence of listeriae in the gravid colon. This latter observation, made possible through the use of the natural oral inoculation route, may warrant further study since, in women, colonic colonization could serve as a valuable predictor of an at-risk pregnancy.

A principal reason for the increased lethality in gravid mice could be the ability of the implanted embryo to serve as a privileged site for bacterial replication should sufficient numbers of circulating bacteria be present to effect colonization [11]. Mouse blastocyst implantation occurs at *ca.* 3.5 gd [9]. Consequently, circulating listeriae could find a protective environment available at approximately this time. Our observation that susceptibility to lethal infection was increased even at 0.5 gd is consistent with this view because listeriae were present in the livers and spleens for periods that overlapped with blastocyst implantation.

The reason that listeriosis lethality decreased later in gestation is not known, but may relate to the presence of a more fully developed placental barrier to infection. Another plausible explanation is that some of these infected individuals gave birth (*i.e.* expelled potentially infective nidi). These factors, and other perinatal events, introduce variables into the interpretation of results at the later gestational stages but do not impact our finding that gravid animals become more prone to lethal infection at a previously unidentified early stage. This finding clearly casts doubt on the role of generalized immune modulation as an explanation for pregnancy's effect on listeriosis susceptibility, at least in mice.

Mice, other rodents [16], and humans have a hemochorial placenta and a similar early placental development [17]. Although several rodent species other than mice have been argued to be more representative of humans [18], [19], all gravid mammalian hosts develop a strikingly high listeriosis susceptibility, and mice are no exception to this rule. Consequently, the magnitude and implication of observations regarding the onset of susceptibility in any model host should be regarded as compelling.

In summary, our findings suggest that listeriosis could be a factor in early pregnancy loss and support the adoption of routine microbiological evaluation of human concepti spontaneously aborted in early pregnancy. Our results also point out that colonic listerial carriage in pregnant women could be an especially sensitive indicator of exposure and deserving of subsequent careful monitoring.

## Materials and Methods

### Ethics Statement

This study was carried out in strict accordance with the recommendations in the Guide for the Care and Use of Laboratory Animals of the National Institutes of Health. The study was approved by the North Carolina State University Institutional Animal Care and Use Committee (Assurance Number: A3331-01).

### Bacterial culture

The mouse oral virulent *L. monocytogenes* serotype 4nonb strains F6214-1 and PAS351 were used in this study [11]. The strains are equally virulent and isogenic except for a Tn917 insertion in PAS351 that confers lincomycin and erythromycin resistance [11]. The strains were used in a 1∶1 mixture. The 1∶1 mixture was used to detect possible infectious bottlenecks [11] in the infectivity experiments. This precaution proved unnecessary and total listerial numbers are presented herein. Bacteria were propagated at 37°C, in brain heart infusion (BHI) broth, or BHI media supplemented with 1.5% agar (Difco). Broth cultures were grown overnight with shaking. The resulting stationary phase culture was subcultured and grown with shaking to logarithmic phase (OD_600_ 0.3–0.6).

### Oral inoculations

Bacteria were harvested by centrifugation and resuspended in phosphate buffered saline (PBS). Viable counts (colony forming units [cfu]) were determined as previously described [11]. Mice were orally inoculated with a 20 µl volume after depriving them of water for 2 h.

### Timed pregnant mice

CD-1 mouse pairings were carried out by housing one male with three females overnight. Pregnant mice were identified by the presence of a postcoital vaginal plug the following morning. Gestational days (gd) were established based on an initial 0.5 gd determination the morning following mating. Sets of four mice were inoculated at 0 [nonpregnant], 0.5, 3.5, 6.5, 9.5, 14.5 gd.

### Fifty percent lethal dose determinations

Groups of at least 4 mice were inoculated with 10^7^, 10^8^ or 10^9^ cfu. Inoculated animals were observed for 11 days. Dead and moribund animals were used to determine the fifty percent lethal dose (LD_50_) for each of the gestational stages [20].

### Liver and spleen infectivity determinations

Infectivity experiments were performed essentially as for the LD_50_ experiments except a single dose (averaging 4×10^8^ cfu) was used in all cases. Mice were sacrificed 96 h post inoculation and the cfu in the liver, spleen, uterus and colonic contents were enumerated as previously described [11].

### Statistical treatment

LD_50_ values were calculated as previously described [11] and subjected to Probit analysis for mean differences using the Minitab 14 statistical analysis program. Median differences involving liver and spleen colonization levels were analyzed by the Mann-Whitney rank sum test using the Minitab 14 statistical analysis program. Binary outcome data in listerial colonic carriage were analyzed for mean differences using Fisher's exact test offered on Vassar Stats. In all cases significance was defined as P<0.05.
